# Pricing of geometric Asian options in the Volterra-Heston model

**DOI:** 10.1007/s11147-025-09211-w

**Published:** 2025-03-09

**Authors:** Florian Aichinger, Sascha Desmettre

**Affiliations:** 1 Institute for Financial Mathematics and Applied Number Theory, Johannes Kepler University of Linz, AT-4040 Linz, Austria; 2 Johann Radon Institute for Computational and Applied Mathematics (RICAM), Austrian Academy of Sciences, AT-4040 Linz, Austria

**Keywords:** Volterra-Heston model, Rough volatility, Asian options, Options pricing, Fourier inversion method, Affine Volterra processes, 45D05, 60B15, 60L20, 91G20

## Abstract

Geometric Asian options are a type of option where the payoff depends on the geometric mean of the underlying asset over a certain period of time. This paper is concerned with the pricing of such options for the class of Volterra-Heston models, covering the rough Heston model. We are able to derive semi-closed formulas for the prices of geometric Asian options with fixed and floating strikes for this class of stochastic volatility models. These formulas require the explicit calculation of the conditional joint Fourier transform of the logarithm of the stock price and the logarithm of the geometric mean of the stock price over time. Linking our problem to the theory of affine Volterra processes, we find a representation of this Fourier transform as a suitably constructed stochastic exponential, which depends on the solution of a Riccati-Volterra equation. Finally, we provide a numerical study for our results in the rough Heston model.

## Introduction

The stochastic volatility model of Heston (1993) is nowadays a standard model for pricing financial derivatives. In contrast to the Black-Scholes model with constant volatility, in the Heston model, the volatility follows itself a stochastic process; in particular, one obtains the following dynamics for the stock price and its volatility under a risk-neutral measure $\mathbb {Q}$:^1^

1.1 $$\begin{aligned} d S_t= & S_t \left( r dt + \sqrt{\nu _t}d B_t^S\right) , \end{aligned}$$

1.2 $$\begin{aligned} d \nu _t= & \kappa (\theta -\nu _t) dt + \sigma \sqrt{\nu _t} d B_t^{\nu }, \end{aligned}$$

where the process $\nu$ with $\nu _0\ge 0$ is a Cox-Ingersoll-Ross process. The constants $\kappa , \theta , \sigma$ are assumed to be positive and satisfy the Feller condition $2\kappa \theta \ge \sigma ^2$, $(B^{\nu }_t)$ and $(B^{S}_t)$ are $(\mathcal {F}_t)$-adapted Brownian motions, where $\mathcal {F}_t:=\sigma (B_u^S,B_u^{\nu }:u\le t)$ such that $\langle B^S,B^{\nu }\rangle _t = \rho t$, and *r* denotes the risk-free interest rate. This model fixes, on the one hand, the problem of a non-constant volatility smile, which the Black-Scholes model cannot produce, and, on the other hand, is more suitable to reproduce stylized facts of financial data such as the leverage effect and the mean reversion of volatility. Most importantly, the Heston model still gives a (semi-)explicit option pricing formula in terms of the solution of a Riccati ordinary differential equation which is crucial for a (fast) calibration to market data.

While in the classical Heston model, the volatility process (1.2) follows a Brownian semi-martingale, this modeling approach was challenged by the much-noticed study (Gatheral et al., 2018). Their analysis of several historical volatility time-series from high frequency data came to the conclusion that for a very wide range of assets, historical volatility exhibits a behavior which is much rougher than that of a Brownian motion. This is in line with the influential paper (Andersen et al., 2003) that demonstrates evidence for fractionally integrated variance by investigating high-frequenvcy intraday data in order to model and forecast realized volatity. These observations were more recently confirmed by other statistical studies such as Fukasawa et al. (2022) and Bennedsen et al. (2022). More precisely, according to Gatheral et al. (2018), the dynamics of the logarithmic volatility is quite similar to that of a fractional Brownian motion (fBm) with Hurst parameter of order 0.1. Recall that the fBm $(B^H_t)_{t \ge 0}$ is a continuous Gaussian process with the properties:

- $$B^H_0 = 0$$, [starts in 0]
- $$\mathbb {E}[B^H_t] = 0 \text{ for } \text{ all } t \ge 0$$, [centered]
- $$\mathbb {E}[B^H_t B^H_s]=\tfrac{1}{2} (|t|^{2H}+|s|^{2H}-|t-s|^{2H})$$. [covariance function]

Here $H \in (0,1)$ is called the Hurst parameter and we have the following characterisation: For $H = 1/2$ we recover the standard Brownian motion $(B_t)_{t\ge 0}$ with independent increments. If $H> 1/2$, increments are positively correlated whereas for $H < 1/2$, increments are negatively correlated. In the Mandelbrot-van Ness representation, the fBm is written as a convolution type integral with respect to a standard Brownian motion *B*:

1.3 $$\begin{aligned} B_t^H=\frac{1}{\Gamma (H+\frac{1}{2})}\int _{-\infty }^0 ((t-s)^{H-\frac{1}{2}}-(-s)^{H-\frac{1}{2}})dB_s + \frac{1}{\Gamma (H+\frac{1}{2})}\int _0^t (t-s)^{H-\frac{1}{2}}dB_s \end{aligned}$$

The fractional Brownian motion has Hölder regularity $H - \epsilon$, hence a smaller Hurst parameter leads to ‘rougher’ paths. These findings have motivated the study of various rough volatility models in the literature. Among these are the rough fractional stochastic volatility model (Gatheral et al., 2018), the rough Bergomi model (Bayer et al., 2016), and the fractional and rough Heston models (Comte et al., 2012; Guennoun et al., 2018; El Euch and Rosenbaum, 2019). While the rough volatility literature has grown into a very active and prolific branch of research within the last years, some authors contested the interpretation of Gatheral et al. (2018). For example Rogers (2023) and Rømer (2022) propose an alternative volatility model driven by two Ornstein-Uhlenbeck processes which can as well explain the statistical observations. Cont and Das (2024) argue that the origin of the roughness observed in realized volatility time series lies in the estimation error rather than the volatility process itself.

In El Euch and Rosenbaum (2019), the popular Heston model (Heston, 1993) was adapted to the rough volatility framework by using a fractional process with Hurst index $H<\frac{1}{2}$ as driver of the volatility process. Thus, the dynamics of the volatility process (1.2) become

1.4 $$\begin{aligned} \nu _t = \nu _0 + \frac{1}{\Gamma (\alpha )} \int _0^t (t-s)^{\alpha -1} \kappa (\theta -\nu _s) ds + \frac{1}{\Gamma (\alpha )} \int _0^t (t-s)^{\alpha -1} \sigma \sqrt{\nu _s} dB^{\nu }_s, \end{aligned}$$

where $\Gamma$ denotes the Gamma-function. The parameter $\alpha \in (\frac{1}{2},1)$ governs the roughness of the paths and is related to the Hurst parameter of the fractional Brownian motion via $\alpha =H+\frac{1}{2}$ (cf. equation (1.3)). As explained in El Euch et al. (2018), this type of rough volatility models appear naturally as scaling limits of microstructural pricing models with self-exciting features driven by Hawkes processes. It turns out that besides retrieving all the statistical stylized facts of volatility, the rough Heston model also reproduces very well the behavior of the implied volatility surface, in particular the at-the-money skew (cf. Bayer et al. (2019)). Of course, the rough Heston model is non-Markovian, i.e. in order to predict the future of $\nu$, one needs to know its entire history. Due to the absence of the semimartingale and Markov properties, many tasks such as option pricing become more challenging in this framework. However, researchers developed new techniques to circumvent these problems. In El Euch and Rosenbaum (2019), the characteristic function of the log-price in the rough Heston model was computed using a link between fractional volatility models and its microstructural foundation. Similar to the classical case, the characteristic function can be calculated (semi-)explicitly in terms of the solution of a fractional Riccati equation.

A more general class of volatility models covering this rough Heston model is obtained by modeling the volatility process as a stochastic Volterra equation of convolution type. In this so called Volterra-Heston model, introduced in Abi Jaber et al. (2019) and Abi Jaber and El Euch (2019), one obtains the following dynamics for the stock price and its volatility under a risk-neutral measure $\mathbb {Q}$:

1.5 $$\begin{aligned} d S_t = r S_t dt + \sqrt{\nu _t} S_t d B_t^S, \end{aligned}$$

1.6 $$\begin{aligned} \nu _t = \nu _0 +\int _0^t \mathcal {K}(t-s)(\kappa (\theta -\nu _s))ds+\int _0^t \mathcal {K}(t-s)\sigma \sqrt{\nu _s}dB_s^{\nu }\,, \end{aligned}$$

with integral kernel $\mathcal {K} \in L^{2}([0,T],\mathbb {R})$. The constants $\kappa , \theta , \sigma$ are again assumed to be positive, $(B^{\nu }_t)$ and $(B^{S}_t)$ are Brownian motions such that $\langle B^S,B^{\nu }\rangle _t = \rho t$, and *r* denotes the risk-free interest rate. We consider a filtration $\{\mathcal {F}_t,t\ge 0\}$ where $\mathcal {F}_t:=\sigma (B_u^S,B_u^{\nu }:u\le t)$. Note that if the kernel $\mathcal {K}$ fulfills

1.7 $$\begin{aligned} \begin{aligned}&\mathcal {K}\in L_{\operatorname {loc}}^2 (\mathbb {R}_+,\mathbb {R}) \text { and there is } \gamma \in (0,2] \text { such that } \int _0^h \mathcal {K}(t)^2 (t) dt = O(h^{\gamma }) \\&\text {and } \int _0^T (\mathcal {K}(t+h)-\mathcal {K}(t))^2 dt = O(h^{\gamma }) \text { for every } T<\infty , \end{aligned} \end{aligned}$$

and the shifted kernels $\Delta _h \mathcal {K}$, where $\Delta _h \mathcal {K}(t):= \mathcal {K}(t+h)$, satisfy the condition

1.8 $$\begin{aligned} \begin{aligned}&\Delta _h\mathcal {K} \text { is nonnegative, not identically zero, non-increasing and continuous}\\&\text {on }(0, \infty ),\text { and its resolvent of the first kind } L \text { is nonnegative and non-}\\&\text {increasing in that } s \mapsto L([s, s + t])\text { is non-increasing for all } t \ge 0, \end{aligned} \end{aligned}$$

then the existence of a unique in law $\mathbb {R}\times \mathbb {R}_+$-valued continuous weak solution $(S,\nu )$ of (1.5),(1.6) is guaranteed by Abi Jaber et al. (2019)[Theorem 7.1] for any initial condition $(S,\nu )\in \mathbb {R}\times \mathbb {R}_+$. In particular this holds for the fractional integral kernels $\mathcal {K}(t-s) = (t-s)^{\alpha -1}/\Gamma (\alpha )$, $\alpha \in (\frac{1}{2},1)$ in the rough Heston model (1.4).

It is well-known that closed-form solutions for the price of geometric Asian options can easily be derived in the Black-Scholes model, see e.g. Boyle and Potapchik (2008). Yet, already in the Black-Scholes model and even more so in more sophisticated asset pricing models as e.g. Heston’s stochastic volatility model, it is very difficult to obtain similar results for arithmetic Asian options, and one usually relies on either approximated prices, respectively on a valuation based on a suitable Monte-Carlo algorithm or other numerical methods. For instance, Geman and Yor (1993) give the Laplace transform of the time-integral of a geometric Brownian motion, which can be used to compute the price of an arithmetic Asian option. Moreover, a suitable PDE method for the pricing of such options can be found in Vecer (2001). In this spirit, in Kemna and Vorst (1990), the closed-form prices of geometric Asian calls (puts) were used as lower (upper) bounds for the prices of arithmetic Asian calls (puts). In this context, for a more extensive list of relevant references, particularly in the framework of Heston’s model for various exotic path-dependent options, we refer to Ballestra et al. (2007); Desmettre and Wenzel (2021) is concerned with the Monte Carlo valuation of discretely sampled arithmetic and geometric average options in the Black-Scholes model and the stochastic volatility model of Heston in high volatility environments. In Kirkby and Nguyen (2020), efficient Asian option pricing techniques are developed for regime switching jump diffusions and stochastic volatility models.

In the classical Heston model, Kim and Wee (2014) derive semi-analytical pricing formulas for Asian options with fixed and floating strikes. First, they establish formulas for the option prices in terms of the conditional joint Fourier transform of the logarithm of the stock price at maturity and the logarithm of the geometric mean of the stock price over a certain time period. This approach is based on methods such as numeraire changes and the Fourier inversion formula, which go back to, e.g., Gil-Pelaez (1951); Geman and Karoui (1995). It is important to note that the proofs do not depend on the underlying volatility model. The joint Fourier transform is then represented explicitly as an exponential affine function of the volatility. Finding this representation in the classical model involves Itô calculus and heavily relies on the Markovianity of the volatility process (1.2).

Concerning option pricing in general Volterra models only a few explicit results exist. The seminal paper (Bayer et al., 2016) shows how the rough fractional stochastic volatility (RFSV) model by Gatheral et al. (2018) can be used to price claims on both the underlying and integrated variance, however, no explicit call price formula is given yet. This is done in He and Lin (2019) for the rough Heston model in the sense of El Euch and Rosenbaum (2019), but not for general Volterra kernels. In the context of the rough Heston model, moment explosion was studied in Gerhold et al. (2019). The next step of complexity is then exotic options, cf. the well-known monograph of Zhang (1998). Using the above-explained forward variance methods along the lines of Keller-Ressel et al. (2019) (cf. Bayer et al. (2023)), Horvath et al. (2020) develop efficient Monte Carlo methods and asymptotic approximations for computing option prices and hedge ratios in models where the log-volatility follows a (modulated) Gaussian Volterra processes. Using kernel-based approximation methods, Chevalier et al. (2022) price American options in the general Volterra Heston model. Friz and Wagenhofer (2023) deal with the pricing and hedging of index options in a rough volatility model, using large deviation methods. To conclude, Tong and Liu (2023); Shi et al. (2023) deal with the numerical and analytical pricing of Basket and Barrier options based on Fourier methods in rough (versions of) Heston models, however once again, not in general Volterra models. The pricing of Asian options, i.e., options where the payoff depends on the mean of the underlying asset over a certain period of time, has yet not been studied for the rough Heston model or the more general Volterra-Heston model.

This paper presents pricing formulas for fixed- and floating-strike Asian options in the Volterra-Heston model (1.5), (1.6). The pricing formulas in terms of the joint Fourier transform, as derived by Kim and Wee (2014) for the classical Heston model, are still valid in the Volterra-Heston model. The challenging part is thus to explicitly calculate this Fourier transform when the volatility of the stock is modeled as a Volterra square-root process given via (1.6). In contrast to the classical case, the process (1.6) is not an Itô process anymore; thus, different techniques must be applied in our case. Linking our problem to the theory of affine Volterra processes developed in Abi Jaber et al. (2019), we show that in the Volterra-Heston model, the joint Fourier transform can be represented as a suitably constructed stochastic exponential in terms of the so-called forward variance process and the solution of a Volterra-Riccati equation. As a main result, we then obtain semi-analytic pricing formulas for both fixed- and floating-strike geometric Asian put and call options in the Volterra- Heston model. These formulas are a generalization of the results derived in Kim and Wee (2014) for the classical Heston model, and we also show that their results can be recovered as a special case of the general Volterra approach, even if the respective representations of the Fourier transform appear to be quite different at first sight. We also provide an extensive numerical study of Asian option prices in the rough Heston model, which is of particular practical interest. Implementing the pricing formulas, we numerically calculate prices for the different types of geometric Asian options for varying strikes, maturities, and roughness levels and compare our results to those of Kim and Wee (2014) in the classical case. We observe that the effect of the roughness on the price is highly dependent on the maturity of the option.

The paper is structured as follows. Section 1 serves as an introduction to the class of Volterra-Heston models. In Sect. 2, we present a different parametrization of the Volterra-Heston model via a family of Markovian processes and demonstrate how this can be used for pricing European options. In Sect. 3, we introduce geometric Asian options and a general pricing approach linked to the conditional joint Fourier transform of the logarithm of the stock price and the logarithm of the geometric mean of the stock price over time. In Sect. 4, we derive an explicit representation of this conditional joint Fourier transform for the Volterra-Heston model. Section 5 provides semi-closed pricing formulas for fixed and floating strike Asian call and put options in terms of the Fourier transform calculated in the previous section. In Sect. 6, we compare our results to those obtained in the classical Heston model. Finally, Sect. 7 features a numerical study of our results for the rough Heston model.

## European option pricing in the Volterra-Heston model

To start, we give a short overview of how pricing formulas for European options can be obtained in the Volterra-Heston model. Based on Fourier inversion methods, it is possible to obtain semi-closed formulas for European options in terms of the characteristic function of the stock price $S_T$. In the classical Heston model (Heston, 1993), determining this characteristic function involves Itô calculus and hence heavily relies on the Markovianity of the processes *S* and $\nu$ given via (1.1) and (1.2). The process $\nu$ defined via (1.6) does not have to be Markovian, in particular, this is clearly the case for fractional integral kernels in the rough Heston model (1.4). Thus, in order to apply Itô calculus, one considers an equivalent parametrization of the Volterra Heston model in terms of the family of forward variance processes

2.1 $$\begin{aligned} \xi _t(T):=\mathbb {E}^{\mathbb {Q}}(\nu _T|\mathcal {F}_t)\,, \end{aligned}$$

indexed by *T* (cf. Gatheral and Keller-Ressel (2019); Keller-Ressel et al. (2019)). It turns out that for fixed *T*, the process $\xi _t(T)$ is again an Itô process. As a result of Keller-Ressel et al. (2019), one can find the dynamics of the forward variance process as follows.

### Lemma 2.1

(Prop. 3.2 Keller-Ressel et al. (2019)) Let $\xi _t(T)=\mathbb {E}^{\mathbb {Q}}(\nu _T|\mathcal {F}_t)$ and $R_{\kappa }$ be the resolvent of $\kappa \mathcal {K}$, i.e.

$$\begin{aligned} R_{\kappa }*(\kappa \mathcal {K})=(\kappa \mathcal {K})*R_{\kappa }=\kappa \mathcal {K}-R_{\kappa }. \end{aligned}$$

Then the dynamics of $\xi _t(T)$ are given by

$$\begin{aligned} d\xi _t(T)=\frac{1}{\kappa }R_{\kappa }(T-t)\sigma \sqrt{\nu _t}dB_t^{\nu } \end{aligned}$$

with

$$\begin{aligned} \xi _0(T)=\nu _0 (1-\int _0^T R_{\kappa }(s)ds)+\theta \int _0^T R_{\kappa }(s)ds. \end{aligned}$$

Note that for the fractional kernel $c\frac{t^{\alpha -1}}{\Gamma (\alpha )}$, the resolvent is given by

2.2 $$\begin{aligned} R(t)=ct^{\alpha -1}E_{\alpha ,\alpha }(-ct^{\alpha }), \end{aligned}$$

where $E_{\alpha ,\beta }(z)=\sum \limits _{n=0}^{\infty }\frac{z^n}{\Gamma (\alpha n +\beta )}$ denotes the Mittag-Leffler function. The forward variance form of the Volterra Heston model in log-price notation under a risk-neutral measure $\mathbb {Q}$ is then given by

2.3 $$\begin{aligned} d\operatorname {log}S_t=(r-\frac{1}{2}\nu _t) dt + \sqrt{\nu _t}dB_t^{S}, \end{aligned}$$

2.4 $$\begin{aligned} d\xi _t(T)= \left\{ \begin{array}{ll} \frac{1}{\kappa }R_{\kappa }(T-t)\sigma \sqrt{\nu _t}dB_t^{\nu } & , t<T \\ 0 & , t\ge T \\ \end{array} \right. ;\,\xi _0(T)=\nu _0(1-\int _0^T R_{\kappa }(s)ds)+\theta \int _0^T R_{\kappa }(s)ds. \end{aligned}$$

Here $R_{\kappa }$ is the resolvent of $\kappa \mathcal {K}$, i.e. $R_{\kappa }*(\kappa \mathcal {K})=(\kappa \mathcal {K})*R_{\kappa }=\kappa \mathcal {K}-R_{\kappa }.$ Note that the variance $\nu _t$ can be recovered from the forward variance process via $\nu _t=\xi _t(t)$. The next theorem then ensures that the characteristic function of the log-spot price in the Volterra Heston model can be determined as a suitably constructed exponential in terms of the forward variance and the solution of a Riccati-Volterra equation:

### Theorem 2.2

(Thm. 3.4 Keller-Ressel et al. (2019)) Let $\phi$ be the solution of the Riccati-Volterra equation

$$\begin{aligned} \phi = \mathcal {K}*(Q(iu,\phi )-\kappa \phi ), \text { with} \end{aligned}$$

$$\begin{aligned} Q(iu,\phi )=-\frac{1}{2}(u^2+iu)+\sigma \rho i u \phi +\frac{\sigma ^2}{2}\phi ^2. \end{aligned}$$

Then the auxiliary process $(M_{\tau })_{0\le \tau \le T}$ defined as

2.5 $$\begin{aligned} M_{\tau }:=\operatorname {exp}\left( iu (\operatorname {log}S_{\tau }+r(T-\tau ))+\int _{\tau }^T\xi _{\tau }(s)Q(iu,\phi (T-s))ds\right) \end{aligned}$$

is a true martingale and the characteristic function $\psi _{\operatorname {log}S_T|\mathcal {F}_t}$ is given by

$$\begin{aligned} \psi _{\operatorname {log}S_T|\mathcal {F}_t}(u)=\mathbb {E}^{\mathbb {Q}}(\operatorname {exp}(iu \operatorname {log}S_T)|\mathcal {F}_t)=\mathbb {E}^{\mathbb {Q}}(M_T|\mathcal {F}_t)=M_t. \end{aligned}$$

### Proof

The proof follows as a special case of Lemma 4.1 for $s=0$ and $w=iu$.

$$\square$$

Applying the general model-independent call price formula that connects the distribution of the stock price with its Fourier transform (cf., e.g., Bakshi and Madan (2000)) and using the above explicit representation of the characteristic function yields the following semi-analytic formula for the European call price in the Volterra Heston model.

### Theorem 2.3

The price *C*(0) of a European call option with strike price *K* and maturity *T* is given by

2.6 $$\begin{aligned} C(0)=S_0 \Pi _1- e^{-rT}K\Pi _2, \end{aligned}$$

where

$$\begin{aligned} \Pi _1=\frac{1}{2}+\frac{1}{\pi }\int _0^{\infty }\operatorname {Re}\left[ \frac{e^{-iu\operatorname {ln}(K)}\psi _{\operatorname {log}S_T}(u-i)}{iu\psi _{\operatorname {log}S_T}(-i)}\right] du, \end{aligned}$$

$$\begin{aligned} \Pi _2=\frac{1}{2}+\frac{1}{\pi }\int _0^{\infty }\operatorname {Re}\left[ \frac{e^{-iu\operatorname {ln}(K)}\psi _{\operatorname {log}S_T}(u)}{iu}\right] du, \end{aligned}$$

and $\psi _{\operatorname {log}S_T}$ is the characteristic function of the logarithmic stock price at time *T*. In the general Volterra Heston model (1.6), the characteristic function $\psi _{\operatorname {log}S_T}$ is given by

2.7 $$\begin{aligned} \psi _{\operatorname {log}S_T}(u)=\operatorname {exp}\left( iu (\operatorname {log}S_0+rT)+\int _0^TQ(iu,\phi (T-s))\xi _0(s)ds\right) , \end{aligned}$$

with

$$\begin{aligned} \xi _0(s)=\nu _0(1-\int _0^s R_{\kappa }(y)dy)+\theta \int _0^s R_{\kappa }(y)dy. \end{aligned}$$

### Proof

The proof for the model-independent pricing formula (2.6) is standard and relies on a combination of changes of measure and an application of the Fourier inversion formula. For the sake of completeness, a detailed derivation is given in the Appendix. The explicit representation (2.7) of the characteristic function follows immediately from Theorem 2.2 for the case $t=0$.

$$\square$$

The result of Theorem 2.3 is a generalization of the result presented in He and Lin (2019) for the rough Heston model to general kernels $\mathcal {K}$, in case of a constant interest rate. In He and Lin (2019), the interest rate can also be a stochastic process.

## Geometric Asian options

Asian options are a type of option where the payoff depends on the mean of the underlying asset over a certain period of time. We distinguish between two different types of such options, namely arithmetic and geometric Asian options. Arithmetic Asian options are options where the payoff depends on the arithmetic mean

$$\begin{aligned} A_{[0,T]}=\frac{1}{T}\int _0^T S_u du, \end{aligned}$$

of the underlying over time. For such options, no explicit closed-form solution is known in the Heston model (cf. Kim and Wee (2014)), which is already the case for the Black-Scholes model. There is a vast literature on the numerical valuation of such arithmetic options in the Heston model (e.g., Vecer (2001); Fouque and Han (2001); Broadie and Kaya (2006)), and therefore we focus on finding explicit formulas for Asian options on the geometric mean.

Denote by $G_{[0,T]}$ the geometric mean of the the stock price $S_t$ over the time period [0, *T*], i.e.

3.1 $$\begin{aligned} G_{[0,T]}=\operatorname {exp}\left( \frac{1}{T}\int _0^T\operatorname {log}S_u du \right) . \end{aligned}$$

There are four types of geometric Asian option contracts: fixed-strike Asian call options, fixed-strike Asian put options, floating-strike Asian call options, and floating-strike Asian put options. The payoffs of fixed strike geometric Asian call and put options with strike *K* and maturity *T* are given respectively by

3.2 $$\begin{aligned} \operatorname {max}\{G_{[0,T]}-K,0\},\quad \operatorname {max}\{K-G_{[0,T]},0\}. \end{aligned}$$

The payoffs of floating strike geometric Asian call and put options with maturity *T* are given respectively by

3.3 $$\begin{aligned} \operatorname {max}\{G_{[0,T]}-S_T,0\},\quad \operatorname {max}\{S_T-G_{[0,T]},0\}. \end{aligned}$$

Starting from a risk-neutral pricing approach, it can be shown (cf. Kim and Wee (2014)) that prices of Asian options can be expressed in terms of the conditional joint Fourier transform $\psi$ of the logarithm of the stock price at maturity and the logarithm of the geometric mean of the stock price over a certain time period

3.4 $$\begin{aligned} \psi _t(s,w) =\mathbb {E}^{\mathbb {Q}}\left[ \operatorname {exp}\left( s \operatorname {log}G_{t,T} + w \operatorname {log}S_T \right) |\mathcal {F}_t\right] , \end{aligned}$$

with

3.5 $$\begin{aligned} G_{t,T}= \operatorname {exp}\left( \frac{1}{T}\int _t^T \operatorname {log}S_u du\right) . \end{aligned}$$

Similarly to the derivation of the formula (2.6) for European options, this pricing approach is again based on changes of measure and the Fourier inversion formula, which go back to Gil-Pelaez (1951); Geman and Karoui (1995) and can be applied independently of the underlying volatility model. The main difficulty is thus to find an explicit representation of the Fourier transform (3.4) in the respective market model. In the classical Heston model, (Kim and Wee, 2014) derive such an explicit representation as an exponential in terms of the solution of a Riccati equation. However, their proof heavily relies on the Markovianity of the classical Heston volatility process.

We want to derive pricing formulas for geometric Asian options in the Volterra-Heston model (1.5), (1.6). Thus, we must find an explicit representation for (3.4) when the volatility is modeled as a (non-Markovian) Volterra square-root process. This will be done in the next section. In Sect. 5, we then present semi-closed pricing formulas for fixed- and floating-strike geometric Asian call and put options.

## The joint conditional Fourier transform

In this section we show that in the Volterra-Heston model (1.5), (1.6), the conditional joint Fourier transform (3.4) can be represented as a suitably constructed exponential in terms of the forward variance and the solution of a Riccati-Volterra equation. We start with the following Lemma, which links our problem to the theory of affine Volterra processes developed in Abi Jaber et al. (2019).

### Lemma 4.1

Define a stochastic process $(M_{\tau })_{0\le \tau \le T}$ as

4.1 $$\begin{aligned} M_{\tau }:= \operatorname {exp}(Y_{\tau }) \end{aligned}$$

with

4.2 $$\begin{aligned} \begin{aligned}&Y_{\tau } = \mathbb {E}^{\mathbb {Q}}\left[ \frac{s}{T}\int _0^T \operatorname {log} S_u du + w\operatorname {log}S_T | \mathcal {F}_{\tau }\right] \\&+ \frac{1}{2}\int _{\tau }^T \left( \phi _1^2(T-u) + 2\rho \sigma \phi _1(T-u) \phi _2(T-u) +\sigma ^2 \phi _{2}^2(T-u)\right) \xi _{\tau }(u)du, \end{aligned} \end{aligned}$$

where $\xi _t(s)=\mathbb {E}^{\mathbb {Q}}[\nu _s|\mathcal {F}_t]$ is the forward variance process, $\phi _1\in L^2([0,T],\mathbb {C})$ is an affine linear function given by

4.3 $$\begin{aligned} \phi _1(t)= s \frac{t}{T}+w, \end{aligned}$$

and $\phi _2\in L^2([0,T],\mathbb {C})$ is the unique solution of the Volterra-Riccati equation

4.4 $$\begin{aligned} \phi _2(t)=\int _0^t \mathcal {K}(t-u)\left[ \frac{1}{2}\left( \phi _1^2(u)-\phi _1(u)\right) -\kappa \phi _2(u)+\frac{1}{2}\left( \sigma ^2\phi _2^2(u)+2\rho \sigma \phi _1(u)\phi _2(u)\right) \right] du \end{aligned}$$

on the interval [0, *T*]. Then for all $s,w\in \mathcal {D}:=\{(s,w)\in \mathbb {C}^2: \operatorname {Re}(s)\ge 0, \operatorname {Re}(w)\ge 0, 0\le \operatorname {Re}(s)+\operatorname {Re}(w)\le 1\}$, the process $(M_{\tau })_{0\le \tau \le T}$ is a true martingale.

### Proof

We aim to write the process $(Y_{\tau })_{0\le \tau \le T}$ in terms of a two-dimensional affine Volterra process in order to apply results for this class of affine stochastic processes studied in Abi Jaber et al. (2019). To this end, we observe that equations (1.5) and (1.6) can be written as

4.5 $$\begin{aligned} \begin{aligned} \begin{pmatrix} \operatorname {log}S \\ \nu \end{pmatrix}_t&= \begin{pmatrix} \operatorname {log}S \\ \nu \end{pmatrix}_0 + \int _0^t \begin{pmatrix} 1 & 0 \\ 0 & \mathcal {K}(t-u) \end{pmatrix} \left[ \begin{pmatrix} r \\ \kappa \theta \end{pmatrix} + \begin{pmatrix} 0 & -\frac{1}{2} \\ 0 & -\kappa \end{pmatrix} \begin{pmatrix} \operatorname {log}S \\ \nu \end{pmatrix}_u \right] du \\&\,\,+ \int _0^t \begin{pmatrix} 1 & 0 \\ 0 & \mathcal {K}(t-u) \end{pmatrix} \sqrt{\nu _u} \begin{pmatrix} \sqrt{1-\rho ^2} & \rho \\ 0 & \sigma \end{pmatrix} \begin{pmatrix} d\tilde{B}_u \\ dB_u^{\nu } \end{pmatrix}, \end{aligned} \end{aligned}$$

where $\tilde{B}$ is a Brownian motion independent of $B^{\nu }$. Defining the matrix valued integral kernel $\varvec{\mathcal {K}}$ as $$\varvec{\mathcal {K}}:=\begin{pmatrix} 1 & 0 \\ 0 & \mathcal {K} \end{pmatrix}$$ with scalar kernel $\mathcal {K}$, we see that $(\operatorname {log}S,\nu )^{\top }$ solves the Volterra equation

$$\begin{aligned} (\operatorname {log}S,\nu )_t^{\top } = (\operatorname {log}S,\nu )_0^{\top } + \int _0^t \varvec{\mathcal {K}}(t-u) b((\operatorname {log}S,\nu )_u^{\top })du + \int _0^t \varvec{\mathcal {K}}(t-u) \sigma ((\operatorname {log}S,\nu )_u^{\top }) d(\tilde{B},B^{\nu })_u^{\top }, \end{aligned}$$

where

$$\begin{aligned} a((\operatorname {log}S,\nu )^{\top }) =\sigma ((\operatorname {log}S,\nu )^{\top })\sigma ^{\top }((\operatorname {log}S,\nu )^{\top })= A^{0} + \operatorname {log}S A^1 + \nu A^2 \end{aligned}$$

with

$$\begin{aligned} A^0= \varvec{0}, A^1=\varvec{0}, \text { and } A^2= \begin{pmatrix} 1 & \rho \sigma \\ \rho \sigma & \sigma ^2 \end{pmatrix}, \end{aligned}$$

as well as

$$\begin{aligned} b((\operatorname {log}S,\nu )^{\top })= b^{0} + \operatorname {log}S b^1 + \nu b^2 \text { with } b^0= \begin{pmatrix} r \\ \kappa \theta \end{pmatrix}, b^1= \begin{pmatrix} 0 \\ 0 \end{pmatrix}, b^2= \begin{pmatrix} -\frac{1}{2} \\ -\kappa \end{pmatrix}. \end{aligned}$$

Thus $(\operatorname {log}S,\nu )^{\top }$ is an affine process in the sense of Abi Jaber et al. (2019). Equations (4.3) and (4.4) can be written as a two-dimensional Volterra-Riccati equation

4.6 $$\begin{aligned} (\phi _1,\phi _2)= (w,0){\varvec{\mathcal {K}}} + \left( \left( \frac{s}{T},0\right) +({\phi _1},\phi _2)B+\frac{1}{2}A(({\phi _1},\phi _2))\right) *{\varvec{\mathcal {K}}} \end{aligned}$$

where

$$\begin{aligned} B=(b^1,b^2) \text { and } A((\phi _1,\phi _2))=((\phi _1,\phi _2)A^1(\phi _1,\phi _2)^{\top },(\phi _1,\phi _2)A^2(\phi _1,\phi _2)^{\top }) \end{aligned}$$

with solution $(\phi _1,\phi _2)\in L^2([0,T],(\mathbb {C}^2)^*)$. Thus, $Y_{\tau }$ can be expressed in terms of the two-dimensional affine Volterra process $(\operatorname {log}S,\nu )^{\top }$ and the solution $(\phi _1,\phi _2)$ of (4.6) as

4.7 $$\begin{aligned} \begin{aligned} Y_{\tau }&= \mathbb {E}^{\mathbb {Q}}\left[ (\frac{s}{T},0)*\begin{pmatrix} \operatorname {log} S \\ \nu \end{pmatrix}(T) + (w,0) \begin{pmatrix} \operatorname {log} S \\ \nu \end{pmatrix}_T\Big |\mathcal {F}_{\tau }\right] \\&+ \frac{1}{2} \int _{\tau }^T (\phi _1,\phi _2)_{T-u}\left[ \mathbb {E}^{\mathbb {Q}}(\nu _u|\mathcal {F}_{\tau }) \begin{pmatrix} 1 & \rho \sigma \\ \rho \sigma & \sigma ^2 \end{pmatrix}\right] \begin{pmatrix} \phi _1 \\ \phi _2 \end{pmatrix}_{T-u}du.\\&= \mathbb {E}^{\mathbb {Q}}\left[ (\frac{s}{T},0)*\begin{pmatrix} \operatorname {log} S \\ \nu \end{pmatrix}(T) + (w,0) \begin{pmatrix} \operatorname {log} S \\ \nu \end{pmatrix}_T \Big |\mathcal {F}_{\tau }\right] \\&+ \frac{1}{2} \int _{\tau }^T (\phi _1,\phi _2)_{T-u}a(\mathbb {E}^{\mathbb {Q}}[(\operatorname {log}S,\nu )^{\top }_u|\mathcal {F}_{\tau }])(\phi _1,\phi _2)_{T-u}^{\top }du, \end{aligned} \end{aligned}$$

where

$$\begin{aligned} a(\mathbb {E}^{\mathbb {Q}}[(\operatorname {log}S,\nu )^{\top }_u|\mathcal {F}_{\tau }])=A^0 + \mathbb {E}^{\mathbb {Q}}[\operatorname {log}S_u|\mathcal {F}_{\tau }]A^1 + \mathbb {E}^{\mathbb {Q}}[ \nu _u|\mathcal {F}_{\tau }]A^2. \end{aligned}$$

Thus Theorem 4.3 of Abi Jaber et al. (2019) for $X=(\operatorname {log}S,\nu )$, $u=(w,0)$ and $f=(s/T,0)$ can be applied to prove that $M_{\tau }=\operatorname {exp}(Y_{\tau })$ is a local martingale. It remains to show that *M* is indeed a true martingale. We note that for $s,w\in \mathcal {D}$ it holds that for $\phi _1$ defined via (4.3), we have $\phi _1\in [0,1]$. Since $\operatorname {Re}(u_2)=0$ and $\operatorname {Re}(f_2)=0$, Theorem 7.1 of Abi Jaber et al. (2019) yields that $\phi _2$ defined via (4.4) has a unique solution on [0, *T*] which satisfies $\operatorname {Re}(\phi _2)\le 0$ and $M_{\tau }$ is a true martingale.

$$\square$$

The following Lemma ensures the martingale property for a type of processes that appears in the proof of Theorem 4.3.

### Lemma 4.2

Let $(P_t)_{t\in [0,T]}$ be a stochastic process given by

$$\begin{aligned} P_t:=\int _0^t f(u)\sqrt{\nu _u}dB_u, \end{aligned}$$

where $f\in C^{\infty }([0,T])$ and *B* is a Brownian motion. Then *P* is a true martingale on [0, *T*].

### Proof

*P* is a local martingale since it is an integral with respect to a Brownian motion. In order to prove that *P* is a true martingale, we show that $\mathbb {E}^{\mathbb {Q}}(\langle P,P \rangle _t)<\infty$ for all $t\in [0,T]$. This is the case since for every $t\in [0,T]$: $\mathbb {E}^{\mathbb {Q}}[\langle P,P \rangle _t] = \mathbb {E}^{\mathbb {Q}}[\int _0^t f^2(u) \nu _u du] \le C \int _0^t \mathbb {E}^{\mathbb {Q}}[\nu _u] du < \infty$.

$$\square$$

Given these auxiliary results, we can now proceed to prove that (3.4) has an explicit representation. It will become evident in the next Section that the condition $s,w\in \mathcal {D}$ on the arguments of $\psi$ does not constitute a restriction for deriving the option pricing formulas.

### Theorem 4.3

Let $(s,w)\in \mathcal {D}$. Then for $t\in [0,T]$, the conditional joint Fourier transform $\psi _t$ defined as

$$\begin{aligned} \psi _t(s,w) =\mathbb {E}^{\mathbb {Q}}\left[ \operatorname {exp}\left( s \operatorname {log}G_{t,T} + w \operatorname {log}S_T \right) |\mathcal {F}_t\right] \end{aligned}$$

is given by

4.8 $$\begin{aligned} \begin{aligned}&\psi _t(s,w) = \operatorname {exp}\bigg (s\left( \frac{T-t}{T}\operatorname {log}S_t + r \frac{(T-t)^2}{2{T}}\right) + w\left( \operatorname {log}S_t + r(T-t)\right) \\&+ \int _t^T \left( \frac{1}{2}\left( \phi _1^2(T-u)-\phi _1(T-u)\right) +\sigma \rho \phi _1(T-u)\phi _2(T-u)+\frac{1}{2}\sigma ^2 \phi _2^2(T-u)\right) \xi _t(u)du\bigg ), \end{aligned} \end{aligned}$$

where $\xi _t(s)$ is the forward variance process, $\phi _1\in L^2([0,T],\mathbb {C})$ is an affine linear function given by (4.3) and $\phi _2\in L^2([0,T],\mathbb {C})$ is the solution of the Volterra-Riccati equation (4.4).

### Proof

Starting from the definition of $\psi _t$ and $G_{t,T}$ in (3.4) and (3.5), we have

$$\begin{aligned} \begin{aligned} \psi _t(s,w)&:= \mathbb {E}^{\mathbb {Q}}\left[ \operatorname {exp}\left( s \operatorname {log}G_{t,T} + w \operatorname {log}S_T \right) |\mathcal {F}_t\right] = \mathbb {E}^{\mathbb {Q}}\left[ \operatorname {exp}\left( s \frac{1}{T}\int _t^T \operatorname {log}S_u du + w \operatorname {log}S_T \right) |\mathcal {F}_t\right] \\&= \operatorname {exp}(-s\frac{1}{T}\int _0^t \operatorname {log}S_u du)\mathbb {E}^{\mathbb {Q}}\left[ \operatorname {exp}\left( s \frac{1}{T}\int _0^T \operatorname {log}S_u du + w \operatorname {log}S_T \right) |\mathcal {F}_t\right] . \end{aligned} \end{aligned}$$

Since for the process *M* defined in (4.1) it holds that

$$\begin{aligned} M_T= \operatorname {exp}\left( s \frac{1}{T}\int _0^T \operatorname {log}S_u du + w \operatorname {log}S_T\right) , \end{aligned}$$

and *M* is a martingale by Lemma 4.1, we have

$$\begin{aligned} \mathbb {E}^{\mathbb {Q}}\left[ \operatorname {exp}\left( s \frac{1}{T}\int _0^T \operatorname {log}S_u du + w \operatorname {log}S_T \right) |\mathcal {F}_t\right] = \mathbb {E}^{\mathbb {Q}}[M_T|\mathcal {F}_t]=M_t, \end{aligned}$$

and thus

4.9 $$\begin{aligned} \begin{aligned} \psi _t(w,s)&= \operatorname {exp}(-s\frac{1}{T}\int _0^t \operatorname {log}S_u du)\operatorname {exp}(\mathbb {E}^{\mathbb {Q}}(w\operatorname {log}S_T +\frac{s}{T}\int _0^T \operatorname {log} S_u du | \mathcal {F}_{t})) \\&\times \operatorname {exp}(\frac{1}{2}\int _{t}^T \left( \phi _1^2(T-u) + 2\rho \sigma \phi _1(T-u) \phi _2(T-u) +\sigma ^2 \phi ^2(T-u)\right) \xi _{t}(u)du). \end{aligned} \end{aligned}$$

We simplify the term $\mathbb {E}^{\mathbb {Q}}(\frac{s}{T}\int _0^T \operatorname {log} S_u du + w\operatorname {log}S_T | \mathcal {F}_{t})$. By (1.5), we can write $\operatorname {log}S_T$ as

$$\begin{aligned} \operatorname {log}S_T = \operatorname {log}S_t + (T-t)r -\frac{1}{2}\int _t^T \nu _u du + \int _t^T \sqrt{\nu _u}dB_u^S. \end{aligned}$$

Thus, for the conditional expectation, we get

$$\begin{aligned} \begin{aligned} \mathbb {E}^{\mathbb {Q}}(\operatorname {log}S_T|\mathcal {F}_t)&= \operatorname {log}S_t + (T-t)r - \frac{1}{2}\mathbb {E}^{\mathbb {Q}}(\int _t^T \nu _u du|\mathcal {F}_t) + \mathbb {E}(\int _t^T \sqrt{\nu _u}dB_u^S|\mathcal {F}_t)\\&= \operatorname {log}S_t + (T-t)r - \frac{1}{2}\int _t^T \xi _t(u) du, \end{aligned} \end{aligned}$$

where we have used the fact that the process $P_x^1:=\int _0^x \sqrt{\nu _u}dB_u^S$ is a martingale by Lemma 4.2. Similarly, for the integrated log-spot price, we have

$$\begin{aligned} \begin{aligned}&\int _0^T \operatorname {log}S_z dz = \int _0^t \operatorname {log}S_z dz + \int _t^T (\operatorname {log}S_t + (z-t)r -\frac{1}{2} \int _t^z \nu _u du +\int _t^z \sqrt{\nu _u}dB_u^S)dz \\&= \int _0^t \operatorname {log}S_z dz+(T-t)\operatorname {log}S_t + \frac{1}{2}(T-t)^2 r - \frac{1}{2}\int _t^T (T-u)\nu _u du + \int _t^T (T-u)\sqrt{\nu _u}dB_u^S, \end{aligned} \end{aligned}$$

where we have used the stochastic Fubini theorem from Veraar (2012). Note that the stochastic Fubini theorem is applicable since

$$\begin{aligned} \int_{t}^{T} {\left( {\int_{t}^{z} | \sqrt {\nu _{u} } |^{2} d\langle B^{S} ,B^{S} \rangle _{u} } \right)^{{1/2}} } dz \le T\left( {\int_{0}^{T} {\nu _{u} } du} \right)^{{1/2}} \end{aligned}$$

is finite a.s. Since $P_x^2:=\int _0^x (T-u)\sqrt{\nu _u}dW_u^S$ is again a martingale by Lemma 4.2, we have

$$\begin{aligned} \begin{aligned} \mathbb {E}^{\mathbb {Q}}(\int _0^T\operatorname {log}S_z dz|\mathcal {F}_t)&= \int _0^t \operatorname {log}S_u du + (T-t) \operatorname {log}S_t + \frac{1}{2}(T-t)^2 r - \frac{1}{2}\int _t^T (T-u)\xi _t(u) du. \end{aligned} \end{aligned}$$

This gives us

4.10 $$\begin{gathered} {\mathbb{E}}^{\mathbb{Q}} \left( {\frac{s}{T}\int_{0}^{T} {\log } S_{u} du + w\log S_{T} |{\mathcal{F}}} \right)_{t} = \frac{s}{T}\int_{0}^{t} {\log } S_{u} du + s\left( {\frac{{T - t}}{T}\log S_{t} + r\frac{{(T - t)^{2} }}{{2T}}} \right) \hfill \\ + w\left( {\log S_{t} + r(T - t)} \right) - \frac{1}{2}\int_{t}^{T} {\left( {s\frac{{T - u}}{T} + w} \right)} \xi _{t} (u)du. \hfill \\ \end{gathered}$$

Note that $s\frac{T-u}{T}+w = \phi _1(T-u)$. Taking the exponential and inserting (4.10) back into (4.9) thus yields

$$\begin{aligned} &\psi _t(s,w) = \operatorname {exp}\bigg (s\left( \frac{T-t}{T}\operatorname {log}S_t + r \frac{(T-t)^2}{2T}\right) + w\left( \operatorname {log}S_t + r(T-t)\right) \\&+ \int _t^T \left( \frac{1}{2}\left( \phi _1^2(T-u)-\phi _1(T-u)\right) +\sigma \rho \phi _1(T-u)\phi _2(T-u)+\frac{1}{2}\sigma ^2 \phi _2^2(T-u)\right) \xi _t(u)du\bigg ), \end{aligned}$$

which completes the proof.

$$\square$$

## Pricing geometric Asian options in the Volterra-Heston model

In this section, we present semi-closed formulas for the prices of geometric Asian call and put options with fixed or floating strikes in the Volterra-Heston model. Combining the general pricing approach that links the price of geometric Asian options to the joint Fourier transform (3.4) with the explicit representation of this joint Fourier transform (4.8) derived in the previous section, we obtain the following results.

### Fixed-strike Asian options

For a fixed-strike geometric Asian call option, i.e., an option with payoff $\operatorname {max}\{G_{[0,T]}-K,0\}$, the price of the option at time *t* is given as follows.

#### Theorem 5.1

The price $C_{[0,T]}(t)$ of a fixed-strike geometric Asian call option with strike *K* and maturity *T* and $t\in [0,T]$ is given by

5.1 $$\begin{aligned} \begin{aligned} C_{[0,T]}(t)&= e^{-r(T-t)+\frac{1}{T}\int _0^t \operatorname {log} S_u du}\\&\qquad \times \left[ \frac{\psi _t(1,0)-K_{t,T}}{2}+\frac{1}{\pi }\int _0^{\infty }\operatorname {Re}\left( \big (\psi _t(1+iz,0)-K_{t,T}\psi _t(iz,0)\big )\frac{e^{-iz \operatorname {log}K_{t,T}}}{iz}\right) dz\right] , \end{aligned} \end{aligned}$$

where

5.2 $$\begin{aligned} K_{t,T}:= K e^{-\frac{1}{T}\int _0^t \operatorname {log} S_udu} \end{aligned}$$

and the Fourier transform $\psi _t$ is given by (4.8).

#### Proof

The formula (5.1), which was originally obtained in [Kim and Wee (2014), Thm. 4.1] for the classical Heston model, holds for every model for which the joint Fourier transform (3.4) is known. It remains to prove that we can apply Theorem 4.3 to obtain the exponential representation (4.8) of $\psi _t$ in the Volterra-Heston model. This is the case since all arguments (*s*, *w*) of $\psi$ that appear in (5.1) fulfill that $s,w\in \mathcal {D}=\{(s,w)\in \mathbb {C}^2: \operatorname {Re}(s)\ge 0, \operatorname {Re}(w)\ge 0, 0\le \operatorname {Re}(s)+\operatorname {Re}(w)\le 1\}$.

$$\square$$

For a fixed-strike geometric Asian put option, i.e., an option with payoff $\operatorname {max}\{K-G_{[0,T]},0\}$, the price of the option at time *t* is given as follows.

#### Corollary 5.2

The price $P_{[0,T]}(t)$ of a fixed-strike geometric Asian put option with strike *K* and maturity *T* and $t\in [0,T]$ is given by

5.3 $$\begin{aligned} \begin{aligned} P_{[0,T]}(t)&= e^{-r(T-t)+\frac{1}{T}\int _0^t \operatorname {log} S_u du}\\&\qquad \times \left[ \frac{K_{t,T}-\psi _t(1,0)}{2}+\frac{1}{\pi }\int _0^{\infty }\operatorname {Re}\left( \big (\psi _t(1+iz,0)-K_{t,T}\psi _t(iz,0)\big )\frac{e^{-iz \operatorname {log}K_{t,T}}}{iz}\right) dz\right] , \end{aligned} \end{aligned}$$

where $K_{t,T}$ is defined in (5.2) and the Fourier transform $\psi _t$ is given by (4.8).

#### Proof

The formula (5.3) follows from (5.1) and the put-call parity (cf. [Kim and Wee (2014), Cor. 4.2]). Theorem 4.3 is again applicable since all arguments (*s*, *w*) of $\psi$ that appear in (5.3) fulfill $s,w\in \mathcal {D}$ and hence (4.8) holds.

$$\square$$

### Floating-strike Asian options

The price of a floating strike geometric Asian call option, i.e., an option with payoff $\operatorname {max}\{G_{[0,T]}-S_T,0\}$, at time *t*, is given as follows.

#### Theorem 5.3

The price $\tilde{C}_{[0,T]}(t)$ of a floating-strike geometric Asian call option with maturity *T* and $t\in [0,T]$ is given by

5.4 $$\begin{aligned} \begin{aligned} \tilde{C}_{[0,T]}(t)&= e^{-r(T-t)} \bigg [\frac{1}{2}\Big (e^{r(T-t)}S_t-e^{(1/T)\int _0^t \operatorname {log}S_u du}\psi _t(1,0)\Big )\\&+ \frac{1}{\pi }\int _0^{\infty }\operatorname {Re}\left( \big (e^{(1/T)\int _0^t \operatorname {log}S_u du}\psi _t(1+iz,-iz)-\psi _t(iz,1-iz)\big )\frac{e^{i(z/T) \int _0^t \operatorname {log}S_u du}}{iz}\right) dz\bigg ], \end{aligned} \end{aligned}$$

where $K_{t,T}$ is defined in (5.2) and the Fourier transform $\psi _t$ is given by (4.8).

#### Proof

The formula (5.4) is derived in [Kim and Wee (2014), Cor. 4.4]). Theorem 4.3 is applicable since all arguments (*s*, *w*) of $\psi$ that appear in (5.4) fulfill $s,w\in \mathcal {D}$ and hence (4.8) holds.

$$\square$$

Again, by the put-call parity, the price of a floating-strike geometric Asian put option, i.e., an option with payoff $\operatorname {max}\{S_T-G_{[0,T]},0\}$, at time *t* is given as follows.

#### Corollary 5.4

The price $\tilde{P}_{[0,T]}(t)$ of a floating-strike geometric Asian put option with maturity *T* and $t\in [0,T]$ is given by

5.5 $$\begin{aligned} \begin{aligned} \tilde{P}_{[0,T]}(t)&= e^{-r(T-t)} \bigg [\frac{1}{2}\Big (e^{(1/T)\int _0^t \operatorname {log}S_u du}\psi _t(1,0)-e^{r(T-t)}S_t\Big )\\&+ \frac{1}{\pi }\int _0^{\infty }\operatorname {Re}\left( \big (e^{(1/T)\int _0^t \operatorname {log}S_u du}\psi _t(1+iz,-iz)-\psi _t(iz,1-iz)\big )\frac{e^{i(z/T) \int _0^t \operatorname {log}S_u du}}{iz}\right) dz\bigg ], \end{aligned} \end{aligned}$$

where $K_{t,T}$ is defined in (5.2) and the Fourier transform $\psi _t$ is given by (4.8).

#### Proof

The formula (5.5) is derived in [Kim and Wee (2014), Thm. 4.3]). Theorem 4.3 is applicable since all arguments (*s*, *w*) of $\psi$ that appear in (5.5) fulfill $s,w\in \mathcal {D}$ and hence (4.8) holds.

$$\square$$

## Consistency with results in the classical Heston model

The representation (4.8) of the Fourier transform (3.4) in the Volterra Heston model which we derive in Sect. 4 is of a different structure than the representation derived in the classical Heston model in Kim and Wee (2014). In this section, we explicitly show that in the case of $\mathcal {K}\equiv 1$, both representations are identical using the calculus of convolutions and resolvents and the theory of Riccati equations. In the following, we will denote the representation (4.8) for the special case $\mathcal {K}\equiv 1$ and $t=0$ as $\psi _0^{VH}(s,w)$ and the classical representation of Kim and Wee (2014) as $\psi _0^{CH}(s,w)$. We start with a detailed overview of the two representations.

### Volterra approach

For $\mathcal {K}\equiv 1$, the approach developed in Sect. 4 leads to

6.1 $$\begin{aligned} \begin{aligned}&\psi _0^{VH}(s,w) = \operatorname {exp}\bigg (s\left( \operatorname {log}S_0 + \frac{rT}{2}\right) + w\left( \operatorname {log}S_0 + rT\right) \bigg )\times \\&\operatorname {exp}\bigg ( \int _0^T \left( \frac{1}{2}\left( \phi _1^2(T-{\tau })-\phi _1(T-{\tau })\right) +\sigma \rho \phi _1(T-{\tau })\phi _2(T-{\tau })+\frac{1}{2}\sigma ^2 \phi _2^2(T-{\tau })\right) \xi _0({\tau })d{\tau }\bigg ), \end{aligned} \end{aligned}$$

where

$$\begin{aligned} \xi _0({\tau })=\mathbb {E}^{\mathbb {Q}}[\nu _{\tau }|\mathcal {F}_0]=\nu _0(1-\int _0^{\tau } R_{\kappa }(y)dy)+\theta \int _0^{\tau } R_{\kappa }(y)dy \end{aligned}$$

is the forward variance for the classical Heston volatility ($\mathcal {K}\equiv 1$) at $t=0$, where the resolvent is given by $R_{\kappa }(y)=\kappa e^{-\kappa y}$, $\phi _1\in L^2([0,T],\mathbb {C})$ is an affine linear function given by

$$\begin{aligned} \phi _1({\tau })= s \frac{{\tau }}{T}+w, \end{aligned}$$

and $\phi _2\in L^2([0,T],\mathbb {C})$ is the solution of the Riccati equation

6.2 $$\begin{aligned} \phi _2({\tau })=\int _0^{\tau } \frac{1}{2}\left( \phi _1^2(y)-\phi _1(y)\right) -\kappa \phi _2(y)+\frac{1}{2}\left( \sigma ^2\phi _2^2(y)+2\rho \sigma \phi _1(y) \phi _2(y)\right) dy. \end{aligned}$$

### Classical approach

The classical approach of Kim and Wee (2014) leads to

6.3 $$\begin{aligned} \psi _0^{CH}(s,w)=\operatorname {exp}(z_0)\times \operatorname {exp}\left( \nu _0 \hat{C}(0) +\hat{D}(0) \right) , \end{aligned}$$

where $\hat{C}$ is the solution of the Riccati equation

6.4 $$\begin{aligned} \hat{C}'(\tau )= -\hat{B}(\tau ) + \kappa \hat{C}(\tau ) - \frac{\sigma ^2}{2}\hat{C}^2(\tau ),\quad \hat{C}(T)=z_4, \end{aligned}$$

with the function $\hat{B}$ given as

6.5 $$\begin{aligned} \hat{B}(\tau )= z_1 (T-\tau )^2 + z_2 (T-\tau ) + z_3, \end{aligned}$$

and $\hat{D}$ is given via

6.6 $$\begin{aligned} \hat{D}'(\tau )= -\kappa \theta \hat{C}(\tau ),\quad \hat{D}(T)=0. \end{aligned}$$

The coefficients $z_0$, $z_1$, $z_2$ and $z_3$ are given by

$$\begin{aligned} z_0=s\left( \operatorname {log}S_0 + r\frac{T}{2} - \frac{\kappa \theta \rho T}{2\sigma }-\frac{\rho }{\sigma }\nu _0\right) + w \left( \operatorname {log}S_0 + rT - \frac{\kappa \theta \rho T}{\sigma } -\frac{\rho }{\sigma }\nu _0 \right) , \end{aligned}$$

$$\begin{aligned} z_1= \frac{s^2 (1-\rho ^2)}{2T^2},\, z_2=\frac{s(2\rho \kappa -\sigma )}{2\sigma T}+\frac{sw(1-\rho ^2)}{T},\, z_3=\frac{s\rho }{\sigma T}+\frac{w(2\rho \kappa -\sigma )}{2\sigma }+\frac{w^2(1-\rho ^2)}{2}, \,z_4=\frac{\rho w}{\sigma }. \end{aligned}$$

### Equality of the two approaches

We now show that both representations are indeed equal, i.e., $\psi _0^{VH}(s,w)=\psi _0^{CH}(s,w)$. As a first step, we use time-inversion to get a different representation of (6.3). Defining $C({\tau }):=\hat{C}(T-{\tau })$, we see that $C(0)=\hat{C}(T)=z_4=\frac{\rho }{\sigma }w$ and

$$\begin{aligned} \begin{aligned} C'({\tau }) =-\hat{C}(T-{\tau })&=B(T-{\tau })-\kappa \hat{C}(T-{\tau })+\frac{\sigma ^2}{2}\hat{C}^2(T-{\tau })\\&= z_1 {\tau }^2 + z_2 {\tau } + z_3 - \kappa C({\tau }) +\frac{\sigma ^2}{2}C^2({\tau }). \end{aligned} \end{aligned}$$

Thus *C* fulfills

6.7 $$\begin{aligned} C({\tau })= \frac{\rho }{\sigma }w + \int _0^{\tau } z_1 y^2 + z_2 y + z_3 - \kappa C(y) + \frac{\sigma ^2}{2}C^2(y) dy. \end{aligned}$$

Similarly, defining $D({\tau })=\hat{D}(T-{\tau })$, we obtain $D(0)=\hat{D}(T)=0$ and

$$\begin{aligned} D'({\tau })=-\hat{D}'(T-{\tau })=\kappa \theta \hat{C}(T-{\tau })=\kappa \theta C({\tau }) \end{aligned}$$

and hence *D* is given by

$$\begin{aligned} D({\tau })=\int _0^{\tau } \kappa \theta C(y)dy. \end{aligned}$$

Therefore we obtain with *C* given via (6.7):

6.8 $$\begin{aligned} \psi _0^{CH}(s,w)=\operatorname {exp}(z_0)\times \operatorname {exp}\left( \nu _0 C(T) +\kappa \theta \int _0^T C({\tau }) d{\tau } \right) . \end{aligned}$$

As a next step, we simplify the convolution appearing in (6.1) to show that

$$\begin{aligned} \psi _0^{VH}(s,w) = \operatorname {exp}\bigg (s\left( \operatorname {log}S_0 + r \frac{T}{2}\right) + w\left( \operatorname {log}S_0 + rT\right) \bigg )\times \operatorname {exp}\left( \nu _0 \phi _2(T)+\kappa \theta \int _0^T \phi _2({\tau })d{\tau }\right) . \end{aligned}$$

To this end, we define

6.9 $$\begin{aligned} Q({\tau }):=\frac{1}{2}\left( \phi _1^2({\tau })-\phi _1({\tau })\right) +\sigma \rho \phi _1({\tau })\phi _2({\tau })+\frac{1}{2}\sigma ^2 \phi _2^2({\tau }). \end{aligned}$$

Thus, we can write

$$\begin{aligned} \psi _0^{VH}(s,w) = \operatorname {exp}\bigg (s\left( \operatorname {log}S_0 + r \frac{T}{2}\right) + w\left( \operatorname {log}S_0 + rT\right) \bigg )\times \operatorname {exp}\left( \int _0^T Q(T-{\tau })\xi _0({\tau })d{\tau }\right) . \end{aligned}$$

The result now follows since

$$\begin{aligned} \int _0^T Q(T-{\tau })\xi _0({\tau })d{\tau }&=\int _0^T Q(T-{\tau })\left( \nu _0 - \nu _0 \int _0^{\tau } R_{\kappa }(z)dz +\theta \int _0^{\tau } R_{\kappa }(z)dz\right) d{\tau }\\&= \nu _0 \int _0^T Q(T-{\tau }) d{\tau } - \nu _0 Q*\varvec{1} *R_{\kappa }(T) + \theta Q*\varvec{1} *R_{\kappa }(T)\\&= \nu _0 \int _0^T Q({\tau }) d{\tau } - \nu _0 \varvec{1}*R_{\kappa }*Q (T) + \theta \varvec{1}*R_{\kappa }*Q (T)\\&= \nu _0\left( \phi _2(T)+\kappa \int _0^T \phi _2({\tau })d{\tau }\right) - \nu _0 \kappa \int _0^T \phi _2({\tau })d{\tau } +\kappa \theta \int _0^T \phi _2({\tau })d{\tau }\\&= \nu _0 \phi _2(T) + \kappa \theta \int _0^T \phi _2({\tau }) d{\tau }. \end{aligned}$$

Here, we have used the commutativity of convolution for the third equality. For the fourth equality, we used the identity $\phi _2=\frac{1}{\kappa }R_{\kappa }*Q$ as well as the fact that $\phi _2(T)=\int _0^{T} Q(\tau ) -\kappa \phi _2(\tau )d\tau$ which follows by (6.2) and definition (6.9). A short proof of this identity is provided in the Appendix. In the next step, we show that the solutions of the Riccati equations (6.7) and (6.2) are related to each other. More precisely, we show that if $C({\tau })$ is the solution of (6.7), then $\phi _2({\tau }):= C({\tau })-\frac{\rho }{\sigma }\phi _1({\tau })$ is the solution of (6.2). This is true since

$$\begin{aligned}&C({\tau })-\frac{\rho }{\sigma }\phi _1({\tau }) = \int _0^{\tau } \frac{1}{2}(\phi _1^2(y)-\phi _1(y))-\kappa (C(y)-\frac{\rho }{\sigma }\phi _1(y))\\&+\rho \sigma \phi _1(y)(C(y)-\frac{\rho }{\sigma }\phi _1(y))+\frac{\sigma ^2}{2}(C(y)-\frac{\rho }{\sigma }\phi _1(y))^2 dy\\&\iff C({\tau })-\frac{\rho }{\sigma }\phi _1({\tau }) = \int _0^{\tau } \frac{1}{2}(\phi _1^2(y)-\phi _1(y))+\frac{\rho \kappa }{\sigma }\phi _1(y)-\frac{\rho ^2}{2}\phi _1^2(y) - \kappa C(y)+\frac{\sigma ^2}{2}C^2(y) dy\\&\iff C({\tau })-\frac{\rho }{\sigma }\phi _1({\tau }) = \int _0^{\tau } z_1 y^2 + z_2 y +z_3 - \frac{\rho s}{\sigma T} - \kappa C(y)+\frac{\sigma ^2}{2}C^2(y) dy\\&\iff C({\tau })-\frac{\rho }{\sigma }(\frac{s{\tau }}{T}+w) = - \frac{\rho }{\sigma }\frac{s{\tau }}{T} + \int _0^{\tau } z_1 y^2 + z_2 y +z_3 - \kappa C(y)+\frac{\sigma ^2}{2}C^2(y) dy\\&\iff C({\tau }) = \frac{\rho }{\sigma }w + \int _0^{\tau } z_1 y^2 + z_2 y +z_3 - \kappa C(y)+\frac{\sigma ^2}{2}C^2(y) dy. \end{aligned}$$

Finally, we combine the results of the two previous steps to obtain

$$\begin{aligned} \psi _0^{VH}(s,w)&= e^{s\left( \operatorname {log}S_0 + r \frac{T}{2}\right) + w\left( \operatorname {log}S_0 + rT\right) } \cdot e^{\nu _0 \phi _2(T)+\kappa \theta \int _0^T \phi _2({\tau })d{\tau }}\\&= e^{s\left( \operatorname {log}S_0 + r \frac{T}{2}\right) + w\left( \operatorname {log}S_0 + rT\right) } \cdot e^{\nu _0 (C(T)-\frac{\rho }{\sigma }(s+w))+\kappa \theta \int _0^T C({\tau })-\frac{\rho }{\sigma }(\frac{s}{T}{\tau }+w) d{\tau }}\\&= e^{s\left( \operatorname {log}S_0 + r \frac{T}{2}\right) + w\left( \operatorname {log}S_0 + rT\right) } \cdot e^{-s\frac{\rho }{\sigma }\nu _0 - w\frac{\rho }{\sigma }\nu _0 - s\frac{\kappa \theta \rho T}{2\sigma }-w\frac{\kappa \theta \rho T}{\sigma }}\cdot e^{\nu _0 C(T)+\kappa \theta \int _0^T C({\tau }) d{\tau }}\\&= e^{s\left( \operatorname {log}S_0 + r\frac{T}{2} - \frac{\kappa \theta \rho T}{2\sigma }-\frac{\rho }{\sigma }\nu _0\right) + w \left( \operatorname {log}S_0 + rT - \frac{\kappa \theta \rho T}{\sigma } -\frac{\rho }{\sigma }\nu _0 \right) } \cdot e^{\nu _0 C(T)+\kappa \theta \int _0^T C({\tau }) d{\tau }}\\&= e^{z_0}\cdot e^{\nu _0 C(T) +\kappa \theta \int _0^T C({\tau }) d{\tau }} = \psi _0^{CH}(s,w). \end{aligned}$$

This is the desired equality. $\square$

## Numerical results

In this section, we present numerical results for fixed- and floating-strike geometric Asian call and put options for the class of fractional Heston models, i.e. we consider the model (1.5), (1.6) for the fractional integral kernel $K(t)=\frac{t^{\alpha -1}}{\Gamma (\alpha )}$. For $\alpha =1$, we recover the classical Heston model, and hence, in this special case, our results correspond to those derived in Kim and Wee (2014). Note that the parameter $\alpha$, which governs the roughness of the paths, is related to the Hurst parameter *H* of the fractional Brownian motion via $H=\alpha -\frac{1}{2}$. As already lined out, since the observation was made that volatility is rough (cf. Gatheral et al. (2018)), the class of rough Heston models with $\alpha \in (\frac{1}{2},1)$, has become of particular theoretical and practical interest, and thus we will put particular emphasis on the impact of the roughness parameter on the option price.

### Useful representation of the option prices

To start off, we summarize the pricing formulas derived in the previous sections for the case $t=0$.

#### Corollary 7.1

For $t=0$, the price of a fixed-strike geometric Asian call option is given by

7.1 $$\begin{aligned} \begin{aligned} C_{[0,T]}(0)&= e^{-rT} \left[ \frac{\psi _0(1,0)-K}{2}+\frac{1}{\pi }\int _0^{\infty }\operatorname {Re}\left( \big (\psi _0(1+iz,0)-K\psi _0(iz,0)\big )\frac{e^{-iz \operatorname {log}K}}{iz}\right) dz\right] , \end{aligned} \end{aligned}$$

the price of a fixed-strike geometric Asian put option is given by

7.2 $$\begin{aligned} \begin{aligned} P_{[0,T]}(0)&= e^{-rT} \left[ \frac{K-\psi _0(1,0)}{2}+\frac{1}{\pi }\int _0^{\infty }\operatorname {Re}\left( \big (\psi _0(1+iz,0)-K\psi _0(iz,0)\big )\frac{e^{-iz \operatorname {log}K}}{iz}\right) dz\right] , \end{aligned} \end{aligned}$$

the price of a floating-strike geometric Asian call option is given by

7.3 $$\begin{aligned} \begin{aligned} \tilde{C}_{[0,T]}(0)&= e^{-rT} \bigg [\frac{e^{rT}S_0-\psi _0(1,0)}{2} + \frac{1}{\pi }\int _0^{\infty }\operatorname {Re}\left( \big (\psi _0(1+iz,-iz)-\psi _0(iz,1-iz)\big )\frac{1}{iz}\right) dz\bigg ], \end{aligned} \end{aligned}$$

the price of a floating-strike geometric Asian put option is given by

7.4 $$\begin{aligned} \begin{aligned} \tilde{P}_{[0,T]}(0)&= e^{-rT} \bigg [\frac{\psi _0(1,0)-e^{rT}S_0}{2} + \frac{1}{\pi }\int _0^{\infty }\operatorname {Re}\left( \big (\psi _0(1+iz,-iz)-\psi _0(iz,1-iz)\big )\frac{1}{iz}\right) dz\bigg ], \end{aligned} \end{aligned}$$

and the conditional joint Fourier transform $\psi _0$ can be written as

7.5 $$\begin{aligned} \begin{aligned}&\psi _0(s,w) = \operatorname {exp}\bigg (s\left( \operatorname {log}S_0 + \frac{rT}{2}\right) + w\left( \operatorname {log}S_0 + rT\right) + \int _0^T \nu _0Q({\tau }) + \kappa (\theta -\nu _0) \phi _2({\tau }) d{\tau } \bigg ), \end{aligned} \end{aligned}$$

where $\phi _2\in L^2([0,T],\mathbb {C})$ is the solution of the Volterra Riccati equation

7.6 $$\begin{aligned} \phi _2({\tau })=\int _0^{\tau } K({\tau }-y)\left( \frac{1}{2}\left( \phi _1^2(y)-\phi _1(y)\right) -\kappa \phi _2(y)+\frac{1}{2}\left( \sigma ^2\phi _2^2(y)+2\rho \sigma \phi _1(y) \phi _2(y)\right) \right) dy, \end{aligned}$$

$\phi _1\in L^2([0,T],\mathbb {C})$ is an affine linear function given by

$$\begin{aligned} \phi _1({\tau })= s \frac{{\tau }}{T}+w, \end{aligned}$$

and *Q* is defined as

$$\begin{aligned} Q({\tau })=Q(\phi _1,\phi _2;{\tau }):=\frac{1}{2}\left( \phi _1^2({\tau })-\phi _1({\tau })\right) +\frac{1}{2}\left( \sigma ^2\phi _2^2({\tau })+2\rho \sigma \phi _1({\tau }) \phi _2({\tau })\right) . \end{aligned}$$

#### Proof

The formulas (7.1), (7.2), (7.3) and (7.4) are immediately obtained by setting $t=0$ in equations (5.1), (5.3) (5.4), and (5.5). For the representation of the Fourier transform $\psi _0$, we first observe that the formula (4.8) evaluated at $t=0$ yields

$$\begin{aligned} \psi _0(s,w) = \operatorname {exp}\bigg (s\left( \operatorname {log}S_0 + r \frac{T}{2}\right) + w\left( \operatorname {log}S_0 + rT\right) \bigg )\times \operatorname {exp}\left( \int _0^T Q(T-{\tau })\xi _0({\tau })d{\tau }\right) . \end{aligned}$$

Substituting $\xi _0({\tau })=\nu _0(1-\int _0^{\tau } R_{\kappa }(y)dy)+\theta \int _0^{\tau } R_{\kappa }(y)dy$ and applying the calculus of convolutions and resolvents to the last term yields

$$\begin{aligned} \begin{aligned} \int _0^T Q(T-{\tau })\xi _0({\tau })d{\tau }&=\int _0^T Q(T-{\tau })\left( \nu _0 - \nu _0 \int _0^{\tau } R_{\kappa }(z)dz +\theta \int _0^{\tau } R_{\kappa }(z)dz\right) d{\tau }\\&= \nu _0 \int _0^T Q(T-{\tau }) d{\tau } - \nu _0 Q*\varvec{1} *R_{\kappa }(T) + \theta Q*\varvec{1} *R_{\kappa }(T)\\&= \nu _0 \int _0^T Q({\tau }) d{\tau } - \nu _0 \varvec{1} *R_{\kappa }*Q (T) + \theta \varvec{1} *R_{\kappa }*Q (T)\\&= \nu _0 \int _0^T Q({\tau }) d{\tau } - \nu _0 \kappa \int _0^T \phi _2({\tau })d{\tau } +\kappa \theta \int _0^T \phi _2({\tau }){\tau }. \end{aligned} \end{aligned}$$

Here, we have used the commutativity of convolution for the third and the identity $\phi _2=\frac{1}{\kappa }R_{\kappa }*Q$ (cf. Lemma 9.1 in the Appendix) for the fourth equality. This now gives us (7.5).

$$\square$$

Note that in contrast to (4.8), the resolvent does no longer appear in the representation (7.5) of $\psi _0$ anymore. This fact is particularly useful in the case of fractional integral kernels appearing in the rough Heston model since we do not have to approximate the Mittag-Leffler function appearing in the resolvent (cf. equation (2.2)). Therefore, this allows us to improve the accuracy as well as the computational efficiency of the implementation of our formulas.

### Concrete results

We now present concrete numerical results for fixed- and floating-strike geometric Asian calls and put option prices at 0. In order to have a benchmark parameter set, analogous to Kim and Wee (2014), we use the calibrated market parameters

$$\begin{aligned}\kappa =1.15,\, \theta =0.348 ,\, \sigma =0.39 \text{ and } \rho =-0.64,\end{aligned}$$

which correspond to the daily averages of the respective implied parameters reported in table IV of Bakshi et al. (1997) as well as their choices

$$\begin{aligned}r=0.05,\, S_0=100 \text{ and } \nu _0=0.09.\end{aligned}$$

Two approximations are carried out for the numerical implementation of the analytic formulas. In order to calculate the Fourier transform (7.5), one first has to numerically solve the Volterra Riccati equation (7.6) for $K(t)=\frac{t^{\alpha -1}}{\Gamma (\alpha )}$, which is done using the fractional Adams method, developed in Diethelm et al. (2002). For a detailed error analysis of the fractional Adams method, we refer to Diethelm et al. (2004). Secondly, the infinite integral appearing in (7.1), (7.3) must be truncated. Here, we take an upper limit of $10^2$. This is in line with the observation made in Kim and Wee (2014), that for higher limits the numerical results remain unchanged.

In Table 1, we present prices for fixed-strike geometric Asian call options for different maturities, strike prices, and roughness levels of volatility. In our study, we investigate the case $\alpha \in (\frac{1}{2},1)$, which is the rough Heston model.

The case, where $\alpha \in (1,\frac{3}{2})$, leading to smoother volatility paths than in the classical model is not considered, since in this case the kernel is not completely monotone. In particular condition (1.8) does not hold and the existence, uniqueness, and affine property is not guaranteed.

We observe the following characteristics: The option price decreases with increasing strike. Moreover, the smaller the maturity, the higher the impact of the strike on the option price. Larger maturities lead to higher option prices across all different strikes. All these observations align with the results of Kim and Wee (2014) for the classical Heston model. Note in particular that for $\alpha =1$ in Table 1, we recover their results presented in [Kim and Wee (2014), Table 5].

In addition to the classical Heston model, we now face a further parameter that has to be taken into account, namely the roughness parameter $\alpha$. We observe that the effect of the roughness depends on the maturity of the option. For smaller maturities, the price of the option increases with the roughness level.

For the fixed-strike put option prices in Table 1, the option price rises with increasing strike and maturity. As is also the case for the corresponding call options, only for very large maturities do prices start to decline slightly. The effect of roughness again changes depending on the maturity of the option. Here, the price of the option again increases with the roughness for smaller maturities. However, we observe the reverse effect for larger maturities, i.e., option prices decrease with increasing roughness levels. This is in line with the fact that time-dependent effects of the roughness are well-established in the context of portfolio optimization theory, where the preference between buying a rough or a smooth stock changes with the time-horizon (cf. Abi Jaber et al. (2021); Glassermann and He (2020); Aichinger and Desmettre (2023)). However, in addition, the prices listed in the corresponding tables are related to each other via the put-call parity

$$\begin{aligned} C_{[0,T]}(0)-P_{[0,T]}(0) = e^{-rT}(\psi _0(1,0)-K). \end{aligned}$$

The value of the Fourier transform $\psi _0$ also depends on the roughness parameter $\alpha$, and hence, the effects of roughness on put and call option prices cannot be expected to be observed on a one-for-one basis. Please note that the put prices have, at the same time, been checked for correctness with the help of this put-call parity.

The results for floating-strike geometric Asian call and put options given in Table 2 exhibit a similar behavior. Larger maturities again lead to higher option prices for both put and call options. As for fixed-strike options, the effect of the roughness depends on the maturity of the option. For our model parameters, we observe that a change in the effect of roughness appears around $T=1$. For maturities $T<1$, rougher volatility paths lead to higher option prices, while the reverse effect can be observed for $T>1$ for both put and call options. Note that in contrast to the fixed-strike case, the stock price at maturity $S_T$, which appears in the payoff, is dependent on the roughness parameter as well. Here, the prices listed in the corresponding tables are related to each other via the put-call parity

$$\begin{aligned} \tilde{C}_{[0,T]}(0)-\tilde{P}_{[0,T]}(0) = S_0 - e^{-rT}\psi _0(1,0), \end{aligned}$$

and have once more been checked for correctness using this parity.

**Table 1:** Fixed-strike geometric Asian call and put option prices for different maturities, strike prices, and roughness levels for model parameters $S_0=100$, $\nu _0=0.09$, $t=0$, $r=0.05$, $\kappa =1.15$, $\theta =0.348$, $\sigma =0.39$

T (years)	K	Call option value			Put option value		
		$\alpha =1.00$	$\alpha =0.75$	$\alpha =0.60$	$\alpha =1.00$	$\alpha =0.75$	$\alpha =0.60$
0.2	90	10.6571	10.8151	10.9546	0.4526	0.6424	0.8061
	95	6.5871	6.7931	6.9556	1.3329	1.5705	1.7575
	100	3.4478	3.6182	3.7484	3.1438	3.3459	3.5005
	105	1.4552	1.5338	1.5976	6.1014	6.2118	6.3000
	110	0.4724	0.4841	0.5016	10.0689	10.1124	10.1542
0.4	90	11.7112	11.9773	12.1707	1.3799	1.7123	1.9469
	95	8.0894	8.3894	8.5983	2.6591	3.0254	3.2755
	100	5.1616	5.4415	5.6345	4.6323	4.9785	5.2128
	105	3.0018	3.2178	3.3695	7.3735	7.6558	7.8489
	110	1.5715	1.7089	1.8103	10.8442	11.0478	11.1906
0.5	90	12.2329	12.5314	12.7380	1.8619	2.2389	2.4909
	95	8.7553	9.0844	9.3050	3.2609	3.6685	3.9345
	100	5.8971	6.2119	6.4211	5.2792	5.6726	5.9273
	105	3.7072	3.9697	4.1460	7.9659	8.3069	8.5288
	110	2.1589	2.3501	2.4823	11.2941	11.5638	11.7417
1	90	14.5779	14.9390	15.1588	4.1801	4.6239	4.8798
	95	11.5551	11.9353	12.1637	5.9135	6.3763	6.6408
	100	8.9457	9.3239	9.5499	8.0602	8.5211	8.7830
	105	6.7559	7.1130	7.3263	10.6266	11.0663	11.3155
	110	4.9723	5.2932	5.4859	13.5991	14.0027	14.2312
1.5	90	16.5030	16.8533	17.0544	6.2606	6.6283	6.8267
	95	13.7625	14.1213	14.3261	8.1588	8.5350	8.7371
	100	11.3374	11.6939	11.8969	10.3725	10.7464	10.9465
	105	9.2245	9.5687	9.7647	12.8983	13.2599	13.4529
	110	7.4122	7.7358	7.9205	15.7246	16.0658	16.2473
2	90	18.0914	18.4083	18.5845	8.0965	8.3323	8.4548
	95	15.5640	15.8803	16.0558	10.0933	10.3285	10.4502
	100	13.2933	13.6034	13.7755	12.3467	12.5758	12.6939
	105	11.2728	11.5719	11.7379	14.8504	15.0684	15.1804
	110	9.4921	9.7761	9.9339	17.5939	17.7968	17.9005
3	90	20.5102	20.7529	20.8857	11.1123	11.0617	11.0298
	95	18.3060	18.5346	18.6598	13.2116	13.1469	13.1073
	100	16.2895	16.5034	16.6208	15.4986	15.4193	15.3717
	105	14.4531	14.6523	14.7617	17.9658	17.8716	17.8159
	110	12.7882	12.9725	13.0740	20.6044	20.4954	20.4316
8	90	24.7980	24.9009	24.9967	18.0809	17.3813	16.9256
	95	23.4852	23.5573	23.6319	20.1198	19.3894	18.9125
	100	22.2483	22.2920	22.3471	22.2345	21.4757	20.9792
	105	21.0827	21.1003	21.1374	24.4204	23.6356	23.1211
	110	19.9840	19.9778	19.9983	26.6733	25.8646	25.3336
12	90	24.1840	24.3052	24.4514	18.9998	18.2823	17.7561
	95	23.2584	23.3520	23.4766	20.8183	20.0731	19.5254
	100	22.3795	22.4472	22.5516	22.6834	21.9124	21.3445
	105	21.5442	21.5878	21.6734	24.5922	23.7971	23.2103
	110	20.7498	20.7710	20.8388	26.5418	25.7243	25.1198

**Table 2:** Floating-strike geometric Asian call and put option prices for different maturities and roughness levels for model parameters $S_0=100$, $\nu _0=0.09$, $t=0$, $r=0.05$, $\kappa =1.15$, $\theta =0.348$, $\sigma =0.39$

	Call option value			Put option value		
T (years)	$\alpha =1.00$	$\alpha =0.75$	$\alpha =0.60$	$\alpha =1.00$	$\alpha =0.75$	$\alpha =0.60$
0.2	3.9925	4.2467	4.3812	3.3015	3.5240	3.6341
0.4	6.4473	6.7508	6.8805	4.9965	5.2336	5.3222
0.5	7.5727	7.8563	7.9658	5.7216	5.9267	5.9908
1	12.5761	12.5965	12.5621	8.5845	8.5222	8.4516
1.5	16.8305	16.5500	16.3745	10.5698	10.2720	10.0988
2	20.5478	20.0250	19.7323	11.9781	11.5364	11.2964
3	26.8612	26.0414	25.5759	13.7228	13.1961	12.8935
8	48.0585	47.0888	46.3269	15.1044	14.9363	14.7224
12	59.2926	58.4512	57.6810	13.8699	13.8658	13.7619

## Conclusion

We provide semi-analytic pricing formulas for fixed- and floating-strike geometric Asian options in the class of Volterra-Heston models, which include the rough Heston model. These formulas are obtained by combining a general pricing approach based on the Fourier inversion theorem with an explicit representation of the conditional joint Fourier transform of the logarithm of the stock price at maturity and the logarithm of the geometric mean of the stock price over a certain time period. In contrast to the classical Heston model, the volatility process in the Volterra-Heston model is no longer Markovian, and hence, we have to apply different techniques to obtain this representation as a suitably constructed stochastic exponential in terms of the solution of a Volterra-Riccati equation. This construction requires a link to the theory of affine Volterra processes developed in Abi Jaber et al. (2019). Our work is an extension of the results of Kim and Wee (2014) for the classical Heston model to the class of Volterra-Heston models. We also implement our pricing formulas and provide a numerical study for the rough Heston model, in which we investigate the influence of the roughness of the volatility process on the option prices.

## Data Availability

Data sharing not applicable-no empirical data is used in this study and no new data is generated other than those presented in the tables and figures.

## Appendix

### Proof of the general pricing approach in Theorem 2.3:

Under a risk-neutral measure ${\mathbb {Q}}$, the price of the European call option is given as

$$\begin{aligned} \begin{aligned} C_0&= e^{-rT}\mathbb {E}^{\mathbb {Q}}(\operatorname {max}(S_T-K,0))\\&= e^{-rT}[\mathbb {E}^{{\mathbb {Q}}}(S_T \mathbbm {1}_{\{S_T>K\}})-\mathbb {E}^{{\mathbb {Q}}}(K \mathbbm {1}_{\{S_T>K\}})]. \end{aligned} \end{aligned}$$

For the second expectation, we get

$$\begin{aligned} \mathbb {E}^{{\mathbb {Q}}}(K \mathbbm {1}_{\{S_T>K\}})= K {\mathbb {Q}}(S_T>K). \end{aligned}$$

Define a probability measure ${\mathbb {Q}}_S$ via ${\mathbb {Q}}_S(A):=\mathbb {E}^{{\mathbb {Q}}}\left( \frac{S_T}{\mathbb {E}^{{\mathbb {Q}}}(S_T)}\mathbbm {1}_A\right)$. Then, for the first expectation we obtain

$$\begin{aligned} \begin{aligned} \mathbb {E}^{{\mathbb {Q}}}(S_T \mathbbm {1}_{\{S_T>K\}})&= \mathbb {E}^{{\mathbb {Q}}}\left( \mathbb {E}^{{\mathbb {Q}}}(S_T)\frac{S_T}{\mathbb {E}^{{\mathbb {Q}}}(S_T)} \mathbbm {1}_{\{S_T>K\}}\right) \\&= \mathbb {E}^{{\mathbb {Q}}}(S_T) \mathbb {E}^{{\mathbb {Q}}}\left( \frac{S_T}{\mathbb {E}^{{\mathbb {Q}}}(S_T)} \mathbbm {1}_{\{S_T>K\}}\right) = e^{rT}S_0 {\mathbb {Q}}_S(S_T>K). \end{aligned} \end{aligned}$$

Thus we have

9.1 $$\begin{aligned} \begin{aligned} C_0&= S_0 {\mathbb {Q}}_S(S_T>K) - e^{-rT} K {\mathbb {Q}}(S_T>K)\\&= S_0 {\mathbb {Q}}_S(\operatorname {log}S_T>\operatorname {log}K) - e^{-rT} K {\mathbb {Q}}(\operatorname {log}S_T> \operatorname {log}K)\\ \end{aligned} \end{aligned}$$

Using the identity

$$\begin{aligned} \begin{aligned} \psi _{\operatorname {log}S_T}^{{\mathbb {Q}}_S}(u)&= \mathbb {E}^{{\mathbb {Q}}_S}(e^{iu \operatorname {log}S_T}) = \mathbb {E}^{{\mathbb {Q}}}\left( e^{iu \operatorname {log}S_T} \frac{d {\mathbb {Q}}_S}{d{\mathbb {Q}}}\right) \\&= \mathbb {E}^{{\mathbb {Q}}}\left( e^{iu \operatorname {log}S_T} \frac{S_T}{\mathbb {E}^{{\mathbb {Q}}}(S_T)}\right) = \frac{ \mathbb {E}^{{\mathbb {Q}}}(e^{iu \operatorname {log}S_T} S_T)}{{\mathbb {E}^{{\mathbb {Q}}}(S_T)}} = \frac{\psi _{\operatorname {log}S_T}^{{\mathbb {Q}}}(u-i)}{\psi _{\operatorname {log}S_T}^{{\mathbb {Q}}}(-i)}, \end{aligned} \end{aligned}$$

an application of the Fourier inversion formula now yields

9.2 $$\begin{aligned} \begin{aligned} {\mathbb {Q}}_S(\operatorname {log}S_T> \operatorname {log}K)&= \frac{1}{2}+ \frac{1}{\pi } \int _0^{\infty } \operatorname {Re}\left[ \frac{e^{-iu \operatorname {log}K}\psi _{\operatorname {log}S_T}^{{\mathbb {Q}}_S}(u)}{iu}\right] du \\&= \frac{1}{2}+\frac{1}{\pi }\int _0^{\infty }\operatorname {Re}\left[ \frac{e^{-iu\operatorname {log}(K)}\psi _{\operatorname {log}S_T}^{{\mathbb {Q}}}(u-i)}{iu\psi _{\operatorname {log}S_T}^{{\mathbb {Q}}}(-i)}\right] du, \end{aligned} \end{aligned}$$

and

9.3 $$\begin{aligned} {\mathbb {Q}}(\operatorname {log}S_T>\operatorname {log}K)= \frac{1}{2}+ \frac{1}{\pi } \int _0^{\infty } \operatorname {Re}\left[ \frac{e^{-iu \operatorname {log}K}\psi _{\operatorname {log}S_T}^{{\mathbb {Q}}}(u)}{iu}\right] du. \end{aligned}$$

Inserting (9.2) and (9.3) into equation (9.1) yields the desired result. $\square$

### Equivalent form of the Riccati-Volterra equation

We derive an equivalent representation for the Riccati-Volterra equations appearing in Sects. 6 and 7.

#### Lemma 9.1

The Riccati-Volterra equation

$$\begin{aligned} \phi = K*(Q(\phi ,f)-\kappa \phi ) \end{aligned}$$

with

$$\begin{aligned} Q(\phi ,f)=\frac{1}{2}\left( f^2-f\right) +\frac{1}{2}\left( \sigma ^2\phi ^2+2\rho \sigma f \phi \right) \end{aligned}$$

has equivalent representation

$$\begin{aligned} \phi = \frac{1}{\kappa }R_{\kappa }*Q(\phi ,f). \end{aligned}$$

#### Proof

We start with the Volterra-Riccati equation

$$\begin{aligned} \phi = K*(Q(\phi ,f)-\kappa \phi ) \end{aligned}$$

and subtract $R_{\kappa }*\phi$ from both sides. This yields

$$\begin{aligned} \phi - R_{\kappa }*\phi&= K*(Q(\phi ,f)-\kappa \phi ) - R_{\kappa }*K *(Q(\phi ,f)-\kappa \phi )\\&= (K - R_{\kappa }*K) *(Q(\phi ,f)-\kappa \phi ) \\&= \frac{1}{\kappa } R_{\kappa } *(Q(\phi ,f)-\kappa \phi ) = \frac{1}{\kappa }R_{\kappa }*Q(\phi ,f) - R_{\kappa }*\phi . \end{aligned}$$

And consequently, we get the equivalent representation

$$\begin{aligned} \phi = \frac{1}{\kappa }R_{\kappa }*Q(\phi ,f). \end{aligned}$$

$$\square$$

## Footnotes

1 For the correct change of measure when starting from the physical measure $\mathbb {P}$, please consult Desmettre et al. (2021).
